# Metabolic rate and climate change across latitudes: evidence of mass-dependent responses in aquatic amphipods

**DOI:** 10.1242/jeb.244842

**Published:** 2022-11-25

**Authors:** Milad Shokri, Francesco Cozzoli, Fabio Vignes, Marco Bertoli, Elisabetta Pizzul, Alberto Basset

**Affiliations:** ^1^ Laboratory of Ecology, Department of Biological and Environmental Sciences and Technologies, University of Salento, S.P. Lecce-Monteroni, 73100 Lecce, Italy; ^2^ Research Institute on Terrestrial Ecosystems (IRET–URT Lecce), National Research Council of Italy (CNR), Campus Ecotekne, S.P. Lecce-Monteroni, 73100 Lecce, Italy; ^3^ Department of Life Science, University of Trieste, Via Giorgieri 10, 34127 Trieste, Italy; ^4^ National Biodiversity Future Center, Palermo 90133, Italy

**Keywords:** Metabolic scaling, Body size, Temperature, Global warming, Species distribution, Thermal tolerance

## Abstract

Predictions of individual responses to climate change are often based on the assumption that temperature affects the metabolism of individuals independently of their body mass. However, empirical evidence indicates that interactive effects exist. Here, we investigated the response of individual standard metabolic rate (SMR) to annual temperature range and forecasted temperature rises of 0.6–1.2°C above the current maxima, under the conservative climate change scenario IPCC RCP2.6. As a model organism, we used the amphipod *Gammarus insensibilis*, collected across latitudes along the western coast of the Adriatic Sea down to the southernmost limit of the species' distributional range, with individuals varying in body mass (0.4–13.57 mg). Overall, we found that the effect of temperature on SMR is mass dependent. Within the annual temperature range, the mass-specific SMR of small/young individuals increased with temperature at a greater rate (activation energy: *E*=0.48 eV) than large/old individuals (*E*=0.29 eV), with a higher metabolic level for high-latitude than low-latitude populations. However, under the forecasted climate conditions, the mass-specific SMR of large individuals responded differently across latitudes. Unlike the higher-latitude population, whose mass-specific SMR increased in response to the forecasted climate change across all size classes, in the lower-latitude populations, this increase was not seen in large individuals. The larger/older conspecifics at lower latitudes could therefore be the first to experience the negative impacts of warming on metabolism-related processes. Although the ecological collapse of such a basic trophic level (aquatic amphipods) owing to climate change would have profound consequences for population ecology, the risk is significantly mitigated by phenotypic and genotypic adaptation.

## Teaser

**Summary:** A marginal temperature increase of 1.2°C under future climate change would have particularly profound effects on the physiology and ecology of larger individuals at lower latitudes.

## INTRODUCTION

Climate change has altered ecological rates by shifting patterns of energy flux in ecosystems (Kraemer et al., 2017; O'Connor et al., 2009; Santini et al., 2016) and energy allocation in species (Brown et al., 2004; Yvon-Durocher et al., 2010). Warming in aquatic ecosystems is already having measurable impacts on animal populations, manifested as changes in phenology (Poloczanska et al., 2013), body mass (Audzijonyte et al., 2020; Gardner et al., 2011) and distributional range (Angilletta, 2009; Parmesan and Yohe, 2003). The underlying mechanisms by which climate affects individuals, populations and ecosystems are thus essential for ecosystem functioning. The metabolic rate, i.e. rate of energy use, of organisms is considered a key parameter in ecology, linking individual organisms to populations and ecosystems through a unified currency of energy (Brandl et al., 2022; Cozzoli et al., 2021; Glazier, 2015). Individual ectotherm metabolic rates tend to increase with increasing temperature, owing to its effect on the kinetic energy of cellular components (Gillooly et al., 2001; but see Clarke and Fraser, 2004), although this trend has an upper thermal limit (Schulte, 2015; Sinclair et al., 2016). Thus, metabolic rate is often one of the first individual traits to respond to climate change (Bruno et al., 2015; Verberk et al., 2016b).

The metabolic theory of ecology (MTE) proposes a mechanistic approach to individual energetics, identifying body mass and temperature as the primary determinants of metabolic rate (Brown et al., 2004; Gillooly et al., 2001). This framework has been applied in ecological studies based on simple physical and biological principles. Accordingly, metabolic rate is expected to scale with body mass with an exponent of 0.75 (Kleiber, 1932; West et al., 1997) and with temperature from 0°C to 40°C in accordance with the Boltzmann–Arrhenius factor with an activation energy of ∼0.65 eV (Arrhenius, 1889; Gillooly et al., 2001). The MTE thus implicitly treats the effects of temperature and body mass on individual metabolism independently (Brown et al., 2004; West et al., 1997). However, several studies have found that the effect of temperature on metabolic rate might not follow a universal mass-independent thermodynamic law (e.g. Carey and Sigwart, 2014; Clarke and Fraser, 2004; Glazier, 2020; Killen et al., 2010; Ohlberger et al., 2012). Both empirical and theoretical studies suggest that the allometric exponent of the relationship between metabolic rate and body mass varies with temperature, meaning that the effects of temperature are body-mass dependent (Bullock, 1955; Glazier, 2005, 2020; Killen et al., 2010; Naya et al., 2018; Precht et al., 1973). Several alternative hypotheses to MTE, e.g. the metabolic-level boundaries hypothesis (Glazier, 2005, 2014), the viscosity hypothesis (Verberk and Atkinson, 2013) and the acclimation hypothesis (Fossen et al., 2019), have been developed to explain the body-mass dependency of temperature effects on individual metabolic rates. The effect of temperature on metabolic rate often depends on an organism's thermal physiology and plasticity, which are affected by body mass, life stage, activity level, predation regime/cues, latitude and local climate (Burton et al., 2011; Forster et al., 2012; Glazier, 2020; Glazier et al., 2020; Leiva et al., 2018; Schulte et al., 2011; Terblanche and Chown, 2006). The optimal physiological responses of ectotherms are seen within their local temperature range (Pörtner and Knust, 2007), outside which organism performance and fitness fall (Angilletta, 2009; Anttila et al., 2013; Schulte, 2015; Vasseur et al., 2014), potentially affecting the long-term fate of the species in terms of body mass (Audzijonyte et al., 2020), distributional range (Hickling et al., 2006; Parmesan and Yohe, 2003) and even survival (Hochachka and Somero, 2002; Verberk et al., 2020).

Thermal tolerance, i.e. the temperature zone in which growth, reproduction and survival can be maintained, varies with body mass (Peralta-Maraver and Rezende, 2021; Pörtner and Gutt, 2016). Larger aquatic ectotherms, which possess higher absolute oxygen demands, might be more susceptible to oxygen limitation because their lower surface-area-to-volume ratio constrains their capacity to extract oxygen from their environment and deliver it to their metabolizing tissues (Leiva et al., 2019; Rubalcaba et al., 2020; see also Jutfelt et al., 2018)*.* Thus, a narrowing of thermal tolerance in larger ectothermic individuals might result from a stronger mismatch between oxygen demand and supply (Pörtner and Knust, 2007; Rubalcaba et al., 2020; Verberk et al., 2016a).

In addition to mass dependency, the metabolic rate response of organisms to temperature might be adaptively adjusted in specific environments through phenotypic plasticity or genotype evolution (Benavente et al., 2022; Kefford et al., 2022; Terblanche et al., 2009). A species' thermal tolerance and metabolism also varies with latitude and between climate zones (Hoffmann et al., 2005; Nati et al., 2021; Terblanche and Chown, 2006). For example, the metabolic cold adaptation (MCA) hypothesis predicts that ectotherm organisms that live at higher latitudes with a mean colder climate may show either a higher metabolic level or a higher sensitivity of the metabolic rate to temperature than organisms at lower latitudes with a warmer climate (Chown and Gaston, 1999; Clarke, 1991, 1993; Terblanche et al., 2009). In addition to mean temperature, local thermal variability represents a strong determinant of an organism's thermal niche, which reflects its metabolic rate (Barria et al., 2018; Gaston et al., 1998; Sunday et al., 2019). The climate variability hypothesis (CVH) predicts that high-latitude species often have broader thermal tolerance and possess higher thermal plasticity owing to their local adaptation to a highly variable climate than species at lower latitudes where climate variation is minimal (Bennett et al., 2019; Peralta-Maraver and Rezende, 2021; Sunday et al., 2011, 2019; but see Seebacher et al., 2015).

It is thus important to assess deviations in the mass dependency of the thermal responses of metabolic rate and how this varies biogeographically in order to predict population responses and potential vulnerability to climate change. Empirical research must seek to understand how the standard metabolic rate (SMR) of individuals across a range of body-mass classes varies with temperature, not only within the local temperature range but also with respect to the more extreme temperatures predicted by future warming scenarios. Although recent studies provide significant insight into the ecological responses of ectotherm populations to temperature (Angilletta, 2009; Clarke and Fraser, 2004; Killen et al., 2010; Schulte, 2015), few studies have considered the effects of realistic IPCC climate change scenarios on conspecific populations across latitudinal gradients down to the lower boundary of a species' distribution range (but see Bestion et al., 2015). To help bridge this knowledge gap, this study aimed to (1) assess the response of the SMR of *Gammarus insensibilis* individuals across a range of body-mass classes (herein corresponding to successive life stages) to current annual temperature variation and to the rise in temperature above the local maximum temperature under a conservative climate change scenario, i.e. RCP2.6 (IPCC, 2014), and (2) evaluate the SMR responses to both current annual climate variation and forecasted temperature scenarios of individuals selected from three populations distributed across a range of latitudes along the Western Adriatic coast, approaching the lowest latitudinal edge of the species' distribution.

## MATERIALS AND METHODS

### The model organism and its distributional range

*Gammarus insensibilis* (Stock, 1966) is an ectotherm Atlantic–Mediterranean species of amphipod living in transitional and coastal waters (Costello and Emblow, 2001). They are important components of aquatic ecosystem trophic webs, feeding mainly on detritus and providing nourishment for secondary consumers (Cozzoli et al., 2022; Nelson, 2011; Shadrin et al., 2022), with a lifespan of 1 year (Gerhardt et al., 2011; Węsławski et al., 2020). The geographical distribution of *G. insensibilis* is mostly centred on Europe, Southern Greece, i.e. the Ntivari lagoon at 37.47°N, being their lower latitudinal limit (see https://www.marlin.ac.uk/species/detail/1142) (Tillin and White, 2017).

### Experimental design

**Fig. 1.**
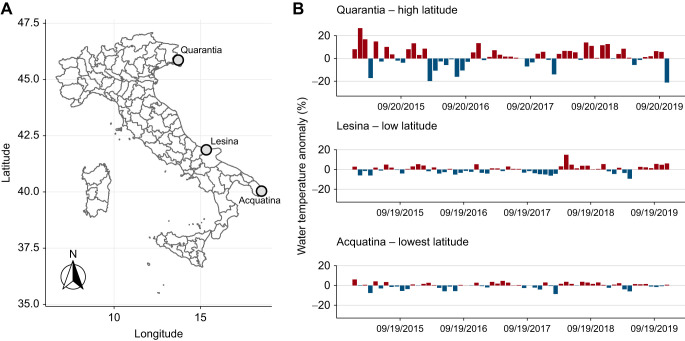
**Specimen collection sites and the water temperature variability at each site.** (A) Map of the study areas and collection sites. (B) Time series of water temperature anomalies at the three collection sites (Quarantia, Lesina and Acquatina lagoons) from 2015 to 2019. The daily water temperature was obtained from the Copernicus Marine Environment Monitoring Service.

The experiment was designed to assess the SMR response to both current annual climate variation and forecasted temperature rises of specimens of *G. insensibilis* belonging to a series of body-mass classes/life stages from a range of geographical areas. *Gammarus insensibilis* specimens were collected simultaneously in autumn from three transitional water bodies along the Western Adriatic coast (Fig. 1A): Quarantia (45.763°N, 13.498°E), Lesina (41.871°N, 15.340°E) and Acquatina (40.444°N, 18.238°E). The three locations were selected to represent a climate gradient approaching the lower latitudinal boundary of its distributional range (Fig. 1, Table 1). The high-latitude collection site, i.e. Quarantia, had relatively lower mean annual water temperature (Table 1) and higher climate variability than the lower-latitude sites, i.e. Lesina and Acquatina (Fig. 1B). The geographical distances between the collection sites were sufficient to assume that the collected specimens belong to distinct populations (Baltazar-Soares et al., 2017; Bayne, 2017).

**Table 1.**
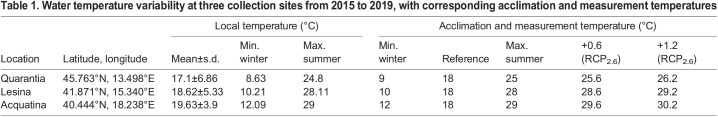
Water temperature variability at three collection sites from 2015 to 2019, with corresponding acclimation and measurement temperatures

The water temperature levels were selected to represent both the annual temperature range currently experienced by each population in accordance with their local climate and the change in temperature predicted to result from climate change (Table 1). To represent the current climate experienced by each of the studied populations, the experimental temperature levels were: (1) the local minimum winter temperature, calculated as the average coldest annual temperature recorded from 2015 to 2019, (2) the reference temperature (set at 18°C for all three sites) and (3) the local maximum summer temperature, calculated as the average warmest annual temperature recorded from 2015 to 2019 (Table 1). We assumed minimal variation in the water column temperature profile, as the waters at the collection sites were relatively shallow (less than 1 m). The daily water surface temperature data for each of the collection sites from 2015 to 2019 were collected by the Copernicus Marine Environment Monitoring Service (CMEMS, 2011; Buongiorno Nardelli et al., 2013) (Table 1). The forecasted temperature levels were chosen with reference to RCP2.6 (IPCC, 2014), i.e. the most conservative climate change scenario, which predicts a mean global water temperature rise of 0.6°C and 1.2°C by 2040 and 2100, respectively (Genner et al., 2017; IPCC, 2014). Although, the RCP2.6 scenario predicts a temperature increase over the mean global temperature, we extended the experimental temperature gradient further by two temperature increments of (4) 0.6°C and (5) 1.2°C above the average current maxima of each collection site (Table 1), in order to estimate the individuals’ metabolic responses and their vulnerability to a relatively narrow temperature increase beyond the local peak temperature that animals experienced.

### Specimen collection and acclimation

At each location, specimens with a range of body masses were collected by scraping the emergent vegetation with 2* *mm mesh metal sieves, and were taxonomically identified with reference to Bellan-Santini et al. (1989, 1998). The body mass of the collected specimens was assumed to reflect different life stages of these moulting amphipods. Herein we thus use ‘small’ and ‘large’ as equivalent to ‘young’ and ‘old’, respectively. After collection, the specimens were transferred to Salento University's Biodiversity and Ecosystem Functioning Laboratory (BIO4IU) in thermo-insulated containers filled with water from the sampling sites and aerated during transport. Authorization for specimen collection was issued by the competent authorities: Friuli Venezia Giulia Regional Administration for the Quarantia site, the Gargano National Park for the Lesina site and the University of Salento for the Acquatina site. The species involved in this study are not endangered or protected. The specimens of each population were kept in the laboratory aquaria at a salinity similar to that of the collection sites (Quarantia 20 PSU, Lesina 22 PSU and Acquatina 21 PSU). They were acclimated to the specific temperatures to be assessed (Table 1) for 2 weeks, which is a sufficient period to minimize any stress and reduce the risk of temperature shock that could severely affect individual metabolic rate (Semsar-kazerouni and Verberk, 2018). Acclimation to temperature levels above and below the collection temperature was achieved at a rate of ±1.5°C day^−1^ in aquaria placed in temperature-controlled environments (KW Apparecchi Scientifici, WR UR series).

Decayed leaves of *Phragmites australis* (Cav.) Trin. ex Steud were supplied as food during the acclimation period in the aquaria and renewed depending on consumption. *Phragmites australis* is known to be one of the largest sources of organic plant detritus in transitional water ecosystems, providing food for macroinvertebrates (Able and Hagan, 2000), including *G. insensibilis* (Cozzoli et al., 2022; Shokri et al., 2021). Before starting the experiment, specimens were sorted by sex under a Nikon stereoscope (SMZ1270). Only males were selected for laboratory experiments because oocyte production in females may induce non-mass-related variability in energy requirements (Glazier et al., 2011).

### SMR setup and measurements

Following Shokri et al. (2019), Glazier and Sparks (1997) and Wrona and Davies (1984), the individual standard metabolic rate (SMR, J day^−1^) was measured as oxygen consumption (*V*_O_2__) of *G. insensibilis* individuals. The animals were kept unfed individually in plastic beakers (200 ml) for 24 h before the SMR measurements at the specific temperatures. To assess their SMR, animals were placed individually in Strathkelvin open-flow system respirometers, also known as flow-through systems (see Fig. S1 for the diagram of the setup). The respirometer measurement system includes a glass water tank (1 litre) filled with the same water as the acclimation aquaria, which was kept magnetically stirred and oxygen-saturated throughout the experiment, using a digital ceramic magnetic stirrer (AREC.X). The stirrer speed was set to 200 rpm, and the operator had observational control over the water tank to avoid supersaturating the water. A peristaltic pump (Watson-Marlow 205 U, 12 channels) provided constant water flow (6 ml h^−1^) to six respirometer chambers (6 ml volume), each containing a single individual. A 0.3* *mm nylon mesh with a nominal outer diameter of 12.07* *mm was placed in each respirometer chamber in order to minimize the individual's spontaneous movement. An equilibration period of 3 h was fixed as the time required to reach a steady concentration of dissolved oxygen, which also enabled specimens to adapt to the respirometer chambers and reduce their spontaneous activity. Upon exiting the chambers, the water was pumped via silicone tubes to the Clark-type microelectrodes (SI1302 Strathkelvin's oxygen electrodes), where the oxygen concentration was measured by an oximeter and recorded and stored using the Strathkelvin software (SI, 929). The operator then read the dissolved oxygen partial pressure (*P*_O_2__, Δtorr) for each individual for 30 min: 15 min for the oxygen concentration curve (in the presence of a specimen: *P*_O_2_,in_), and 15 min for the blank (in the absence of specimens: *P*_O_2_,out_) (see Fig. S2 for the oxygen traces of the three body-mass classes of model organisms). The electrodes were calibrated weekly at the base, i.e. zero calibration with a zero-oxygen solution (2% solution of sodium sulphite in distilled water), and daily to high calibration with air-saturated water (100%). After every experimental trial, we sterilized the respirometer system parts, including the respirometer chambers and silicone tubes, using an autoclave (Hiclave HV) to prevent any possible microbial growth.

The oxygen consumed by each individual *V*_O_2__ (μmol O_2_ h^−1^) was calculated as:
(1)

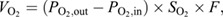
where *P*_O_2,out__ is the partial pressure (torr) of dissolved oxygen in the outflow water of the blank (without specimens), *P*_O_2,in__ is the dissolved oxygen partial pressure (torr) of the respirometer chamber (with a specimen), *F* is the water flow rate (l h^−1^) and *S*_O_2__ is the solubility coefficient of dissolved oxygen in water (μmol l^−1^ torr^−1^). For each temperature and salinity, the solubility coefficient of dissolved oxygen (*S*_O_2__) was obtained from a Loligo oxygen converter (https://www.loligosystems.com/convert-oxygen-units). The rate of oxygen consumption was then converted to metabolic rate (J day^−1^) using an oxyjoule equivalent of 0.45 J (μmol O_2_)^−1^ (Gnaiger, 1983), and by multiplying the resulting value by 24 h. After metabolic measurement, the animals were dried individually in an oven at 60°C for 72 h and then weighed on a micro balance (Sartorius MC5) to the nearest ±0.001 mg.

### Statistical analysis

The differences between the populations collected at each collection site in terms of dry body mass (*M*, mg) and SMR (J day^−1^) were analysed using one-way ANOVA. A linear mixed ANCOVA was used on the complete dataset to investigate the variation of individual SMR with body mass (*M*, mg), temperature range (°C) and collection site. Because the relationship between SMR and body mass is commonly formulated as a power law (Glazier, 2021), they were both log-transformed. The linear mixed model was fitted with full interaction between explanatory variables, i.e. body mass, temperature and collection site, and simplified via a stepwise elimination procedure. The relative importance of the explanatory variables was then assessed by the LMG metric (*R*^2^ partitioned by averaging over orders) (Lindeman et al., 1980). Additionally, we applied an ANCOVA to the linear relationship between log-log transformed SMR and body mass to compare the mass scaling exponents and intercept variation across temperature levels at each collection site. The mass scaling exponents and intercepts of metabolic rate at the different temperatures were all compared with the scaling recorded for the lowest temperature at each collection site.

Moreover, in order to thoroughly assess the thermal response of the mass-specific SMR (J day^−1^ mg^−1^) of individuals of differing body masses to current annual variation and forecasted increases, we placed individuals in three classes using body mass distribution quantiles (0.33, 0.66 and 0.99), corresponding to small (mean±s.d.=1.71±0.51 mg), medium (3.76±0.64 mg) and large (7.36±1.9 mg). Following Brown et al. (2004), we regressed the logarithm of mass-specific SMR (J day^−1^ mg^−1^) on inverse temperature (1/*kT*), where *k* is Boltzmann's constant (8.167×10^−5^ eV K^−1^) and *T* is the temperature in Kelvin. This enabled linear regression of the data, the scaling exponent of the regression quantifying the temperature dependency of mass-specific SMR as an activation energy (*E*, eV). Multiple linear regression was used to analyse the variation of mass-specific SMR with inverse temperature (1/*kT*) and collection site within each body-mass class. The multivariate model was fitted with full interaction between explanatory variables and then simplified via a stepwise elimination procedure. In addition, we estimated the temperature coefficients (*Q*_10_) for each temperature with respect to the minimum temperature across body-mass classes and collection sites (see Fig. S3). The temperature anomaly was calculated as the variation from the average daily temperature for each site, relative to the last 10 years. The significance threshold level was set at *P*=0.05. The analyses were performed in R (https://www.r-project.org/), with the additional packages lme4 (Bates et al., 2015), dplyr (https://CRAN.R-project.org/package=dplyr), relaimpo (Groemping, 2006), Tidyverse (Wickham et al., 2019) and Lubridate (Grolemund and Wickham, 2011).

## RESULTS

### Preliminary data analysis

Overall, we measured 375 male *G. insensibilis* individuals ranging from 4.74 to 15.90* *mm in body length (mean±s.d.=10.25±2.27* *mm) and from 0.4 to 13.57 mg in dry body mass (4.27±2.65 mg). The body mass (*M*, mg) distribution did not differ significantly among the three studied *G. insensibilis* populations, nor did it differ across temperature levels for each population.

### Mass scaling SMRs across temperature and latitudes

Overall, 56.4% of individual SMR variance was explained by the continuous variables, i.e. body mass and temperature, and by the categorical variable, i.e. collection site (Table 2). Individual SMR increased with both temperature (*P*<0.001, 25.3% of explained variance in SMR) and body mass (*P*<0.001, 25.2% of explained variance in SMR), while 4.1% of the observed variance in SMR was explained by the negative interaction between body mass and temperature (*P*<0.01; Table 2), which implies that the SMR response to temperature decreased with increasing body mass. Across collection sites, the SMR level (intercept) was higher for the higher-latitude population than the lower-latitude populations (*P*<0.05; Table 2).

**Table 2.**
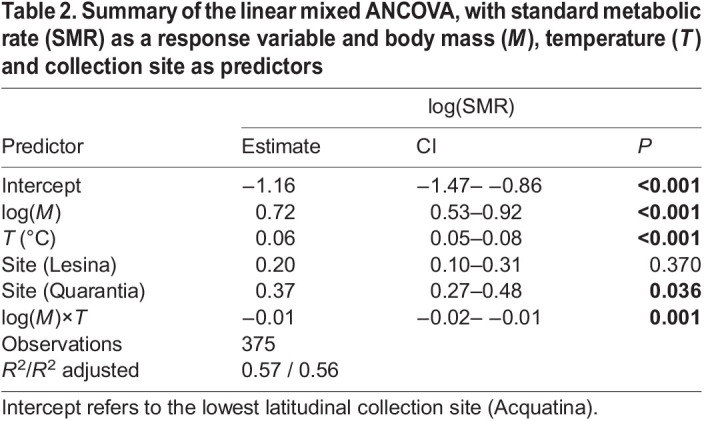
Summary of the linear mixed ANCOVA, with standard metabolic rate (SMR) as a response variable and body mass (*M*), temperature (*T*) and collection site as predictors

**Fig. 2.**
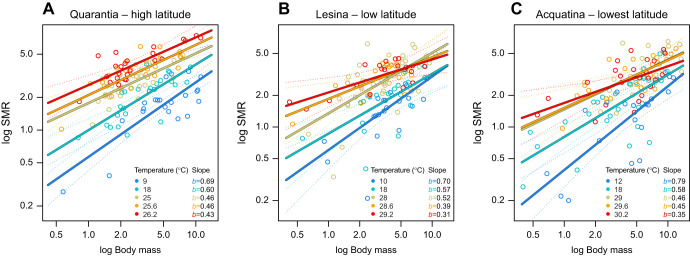
**Standard metabolic rate (SMR; J** **day^–1^) in relation to dry body mass (*M*, mg) across temperature levels at each collection site.** (A) Quarantia, high-latitude population; (B) Lesina, low-latitude population; (C) Acquatina, lowest-latitude population. *b* represents the scaling exponent (slope) of the metabolic rate against body mass for each temperature level.

In the high-latitude population of Quarantia, the estimated intercepts of SMR against body mass increased significantly with temperature from the current minimum to the additional 0.6°C above the current maximum, i.e. 25.6°C, with no significant difference in scaling exponents (slopes) compared with the minimum temperature (Fig. 2A, Table 3). However, at the highest temperature level (26.2°C), which was 1.2°C above the current maxima, the scaling exponent (slope) of metabolic rate against body mass was significantly lower than it was at the minimum temperature (Fig. 2A, Table 3; see Table 4 for the linear equations of the relationship between SMR and body mass across temperatures).

**Table 3.**
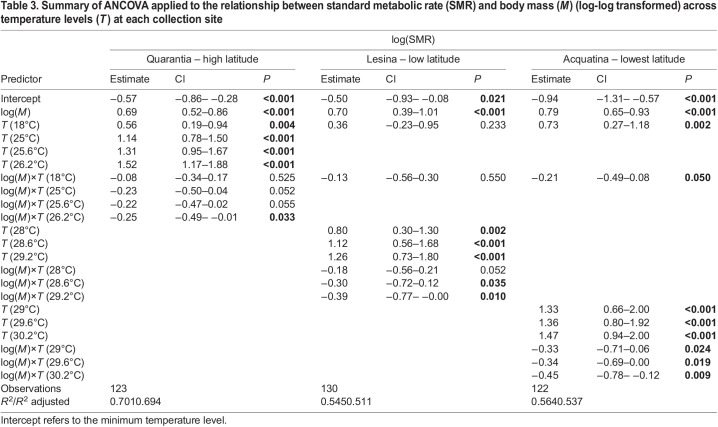
Summary of ANCOVA applied to the relationship between standard metabolic rate (SMR) and body mass (*M*) (log-log transformed) across temperature levels (*T*) at each collection site

**Table 4.**
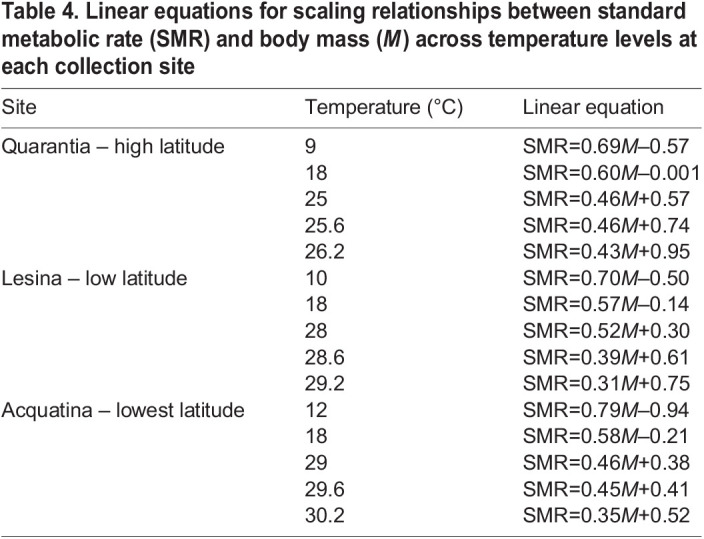
Linear equations for scaling relationships between standard metabolic rate (SMR) and body mass (*M*) across temperature levels at each collection site

In the lower-latitude population of Lesina, the scaling intercepts of metabolic rate against body mass increased significantly with temperature, up to the current maximum (28°C), compared with the minimum temperature, with no significant difference in scaling exponents within the current temperature range (Fig. 2B, Table 3). However, at temperatures above the current maximum (28.6 and 29.2°C), the mass scaling exponents of SMR significantly decreased with respect to the minimum temperature (Fig. 2B, Table 3; see Table 4 for the linear equations of the mass scaling metabolic rate across temperatures).

At the lowest-latitude population, i.e. Acquatina, the scaling exponents of metabolic rate against body mass significantly decreased with temperature with respect to the minimum temperature (12°C) in all cases (Fig. 2C, Table 3; see Table 4 for the linear equations of the relationship between SMR and body mass across temperatures).

### The thermal sensitivity of mass-specific SMR across body-mass classes and latitudes

#### Current annual climate

**Fig. 3.**
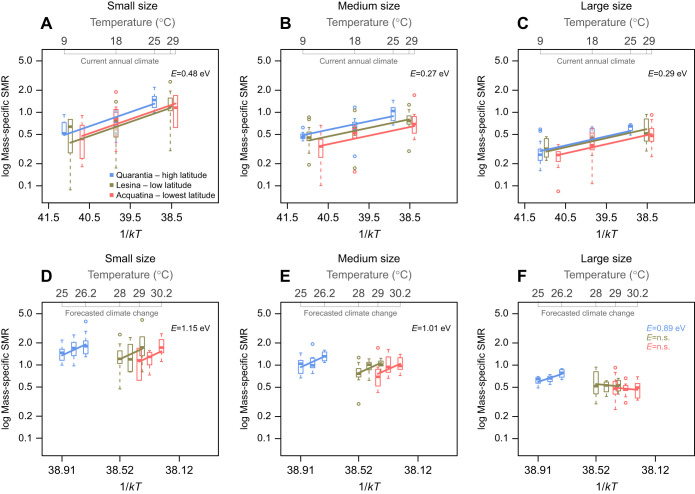
**The thermal responses of mass-specific metabolic rate across body-mass classes and latitudes under current annual climate (minimum to maximum temperature) and forecasted climate change (0.6–1.2°C above the current maxima).** (A–C) Relationship between mass-specific SMR (J day^–1^ mg^–1^) on a logarithmic scale and inverse temperature (1/*kT*), for small (A), medium (B) and large (C) body-mass classes, under current annual climate conditions. (D–F) Relationship between mass-specific SMR on a logarithmic scale and inverse temperature (1/*kT*), for small (D), medium (E) and large (F) body-mass classes under the forecasted climate change scenario. Different colours represent the different populations by location, the boxes show the first, median and third quantiles, and the error bars represent the minimum and maximum values. *E* represents the activation energy [scaling exponent of mass-specific metabolic rate against (1/*kT*)]. Where the activation energies of different collection sites were not significantly different within each body-mass class, we report the pooled *E*. Note that the *x*-axis with the (1/*kT*) values is inverted, reflecting actual temperature, shown at the top of each graph.

Within the current annual temperature range (minimum to maximum temperature), the rate of change of mass-specific SMR with temperature was positive and similar across populations within each body-mass class (Fig. 3A–C, Table 5). However, within each body-mass class, the estimated intercept of the relationship of mass-specific SMR with temperature was significantly higher for the higher latitude population (Quarantia) than for the lower latitude populations (Lesina and Acquatina) (Fig. 3A–C, Table 5). Therefore, at the same temperature, high-latitude individuals had higher SMR per unit of mass than lower-latitude individuals. At all sites, the mass-specific SMR of small individuals increased with temperature, with a scaling exponent equivalent to an activation energy of *E***=**0.48 eV (Fig. 3A, Table 5). The scaling exponent of the relationship between mass-specific SMR and temperature for medium-sized (*E***=**0.27 eV) and large individuals (*E***=**0.29 eV) was lower than it was for small individuals (ANCOVA: *F*_2,235_=39.76, *P*<0.001) (Fig. 3B,C, Table 5; see Fig. S3 for the *Q*_10_ across body-mass classes).

**Table 5.**
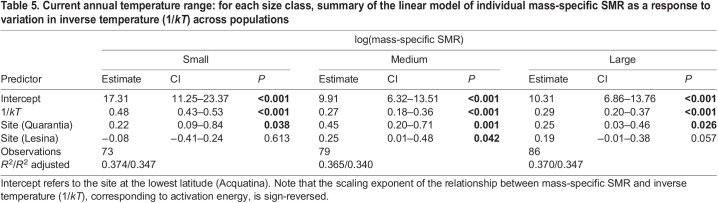
Current annual temperature range: for each size class, summary of the linear model of individual mass-specific SMR as a response to variation in inverse temperature (1/*kT*) across populations

#### Forecasted climate change

Within the forecasted temperature rise (0.6–1.2°C above the current maxima), the mass-specific SMR of the small and medium individuals increased significantly in response to temperature with a similar scaling exponents across latitudes (*E*=1.15 eV for small, and *E*=1.01 eV for medium), and a significantly higher scaling intercept in the high-latitude population than in the lower-latitude populations (Fig. 3D,E, Table 6). However, the mass-specific SMR of large individuals responded differently across latitudes to the forecasted temperature rises. The mass-specific SMR of large individuals from the high-latitude population increased significantly with temperature (*E*=0.89 eV) (similar trend to what was observed for current annual temperature variation), but the thermal response of mass-specific SMR among large individuals from the two lower-latitude populations did not. Indeed, it declined, albeit not significantly (Fig. 3F, Table 6; see Fig. S3 for the *Q*_10_ across body-mass classes).

**Table 6.**
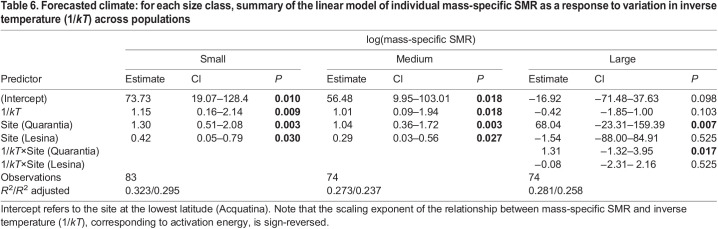
Forecasted climate: for each size class, summary of the linear model of individual mass-specific SMR as a response to variation in inverse temperature (1/*kT*) across populations

## DISCUSSION

We found that temperature altered the mass dependency of metabolic rate, with the mass scaling exponents of metabolic rate decreasing with temperature similarly across latitudes. We observed that the thermal response of the mass-specific SMR of *G. insensibilis* is also body-mass/life-stage dependent, with a stronger response (higher activation energy) in small (young) individuals than in medium and large (old) individuals. Within the current annual temperature range, the rise in mass-specific SMR with temperature was consistent across *G. insensibilis* populations within each body-mass class, with a higher mass-specific SMR level (intercept) in the high-latitude population. In contrast, at temperatures reflecting the forecasted climate scenario, although these were higher than the current maxima by only a small margin, i.e. 0.6°C and 1.2°C, we observed clear geographical variation in the mass-specific SMR response of large individuals. As was observed for the current annual climate, under the forecasted conditions, the mass-specific SMR of large individuals from the high-latitude population increased with temperature rise. However, for large individuals from the lower-latitude populations, towards the lower latitudinal edge of the geographical distribution of *G. insensibilis*, the mass-specific SMR in response to the forecasted climate change declined.

### Mass scaling SMRs across temperature and latitudes

We observed that the mass scaling exponents of SMR decreased as temperature rose. This means that larger and older individuals showed a lesser response to temperature than small individuals. This finding is consistent with both observational data (Daufresne et al., 2009; Pauly, 2010; Precht et al., 1973; Rao and Bullock, 1954) and experimental research (Carey and Sigwart, 2014; Glazier, 2020; Hoefnagel and Verberk, 2015), showing that the effect of temperature on SMR is mass dependent. This is in line with the metabolic-level boundaries hypothesis (*sensu* Glazier, 2005, 2014, 2020), which predicts that the elevation (intercept) of metabolic scaling relationships should increase in response to temperature, whereas the scaling exponent (slope) should decrease owing to changes in the relative influence of surface-area- and volume-related metabolic processes (Glazier, 2005, 2014, 2020). Accordingly, volume-related tissue demand should mainly influence SMR at low metabolic levels, but surface-related resource supply and waste removal should mostly influence SMR at high metabolic levels (Glazier, 2020). It has also been suggested that cold temperatures increase water viscosity and hence the thickness of the boundary layer enveloping the respiratory surfaces of ectotherms, which can result in lower metabolic rates (Verberk and Atkinson, 2013). This effect is expected to be stronger in small individuals, which are more sensitive to increased viscosity and have more difficulty ventilating at low temperatures than larger individuals (Verberk and Atkinson, 2013). Furthermore, it has been hypothesized that organisms cope with the effect of temperature by upregulating or downregulating metabolic rates when growing in cold or warm environments, respectively (Fossen et al., 2019). All of these hypotheses, which are supported by several studies (Gjoni et al., 2020; Glazier, 2020; Killen et al., 2010; Kordas et al., 2022; Lindmark et al., 2018), suggest that temperature effects on the mass dependency of individual metabolism may be widespread in nature.

We observed substantial overall individual variation in SMR, as reported in many other studies of amphipods (Glazier, 2020; Glazier et al., 2011; Semsar-kazerouni and Verberk, 2018; Shokri et al., 2019). This might be due to experimental error, intrinsic factors such as cell size/number (Glazier, 2022), and the effects of ecology or specimens' lifestyles (Careau et al., 2008; Cozzoli et al., 2020; Killen et al., 2016).

### The thermal sensitivity of mass-specific SMR across body-mass classes and latitudes

#### Current annual climate

In contrast to the MTE, which predicts a relatively constant thermal sensitivity of metabolic rate for all body masses within a range of 0–40°C, we observed that the thermal responses of mass-specific SMRs were dependent on individual body mass and life stage. Within current temperature variation, temperature-induced increases in mass-specific metabolic rates were less pronounced in large (*E*=0.29 eV) and medium individuals (*E*=0.27 eV) than in small individuals (*E*=0.48 eV). This is in line with several empirical studies showing that the thermal sensitivity of metabolic rate decreases with body mass and life stage, e.g. in fish (Job, 1957; Silva-Garay and Lowe, 2021), invertebrates (Kordas et al., 2022; Schwartzkopff, 1955; Vernberg, 1959) and planktonic crustaceans (Fossen et al., 2019). Moreover, our observations highlighted geographical variation in mass-specific SMR within each body-mass class under current annual temperatures, with individuals from the high-latitude population, which experienced a more variable climate (see Fig. 1B), showing elevated mass-specific metabolic rates (higher intercepts). In line with our observations, several experimental studies have shown that organisms living in a more variable climate have higher metabolic rates (Magozzi and Calosi, 2015; Sokolova and Pörtner, 2003). Other studies have shown that populations inhabiting a higher latitude often with a colder climate also have higher metabolic rates than populations living at lower latitudes with a warmer climate (Chown et al., 1997; James, 1970; White et al., 2012; see also Terblanche et al., 2009).

#### Forecasted climate change

At temperatures reflecting global warming, our measurements showed that the thermal response of the mass-specific SMRs of *G. insensibilis* varied in accordance with body mass/life stage and geographical distribution. As was observed under current climate conditions, the mass-specific SMR of small (*E*=1.15 eV) and medium-sized individuals (*E*=1.01 eV) of all populations increased in response to a 1.2°C rise above the current maximum summer temperature.

However, the response of large individuals to the forecasted temperature rise differed with latitude. Among large individuals from the lower-latitude populations, i.e. those inhabiting areas near the lower latitudinal boundary of the species' distributional range, the mass-specific SMR declined and reached the peak of their performance at temperatures above the current maxima. The inability of large individuals in the lower-latitude populations to raise their metabolic rate in line with temperature beyond the current annual maximum illustrates the functional limits of their metabolic enzymes. This indicates that future warming under more extreme scenarios could impair the performance of such enzymes, threatening the integrity of membranes, leading to a decline in metabolic rates and thereafter organism death (DeLong et al., 2017; Schulte, 2015).

Our finding also underlines that larger individuals inhabiting the lower latitudinal boundary of their distributional range were subject to metabolic homeostasis (*sensu* Precht et al., 1973), in which their metabolic rates were independent of temperature beyond the current local maxima. Several studies have shown that when facing changes in temperature, often beyond that of their usual environment (Coggins et al., 2021), metabolic homeostasis might occur in aquatic ectotherms (Bullock, 1955; Fangue et al., 2009; Precht et al., 1973; Seibel et al., 2007; Young, 1979). This results in a degree of homeostasis at the cellular level, enabling organisms to maintain their functions in spite of temperature change (Precht et al., 1973). Homeostasis mechanisms are likely to occur where metabolically important substances would become a limiting factor following a rise in metabolic rates with temperature (Precht et al., 1973; Young, 1979). In aquatic organisms, the limiting factor of metabolic rate at the upper thermal limit is the availability of oxygen (Precht et al., 1973; Verberk et al., 2016b), because respiratory demands increase with temperature while oxygen availability and oxygen transport efficiency drop (Boardman and Terblanche, 2015; Verberk et al., 2016b). At high temperatures or under intense physical activity, in aquatic ectotherms, the oxygen supply can no longer meet the increase in oxygen demand, at which point the metabolic rate approaches a limit and its sensitivity to temperature is reduced (Rubalcaba et al., 2020). Because of their higher absolute oxygen demands, larger aquatic ectotherm individuals are likely to experience greater oxygen limitation at high temperatures, which thus reduces aerobic scope and lowers the thermal limit (Atkinson et al., 2006; Lindmark et al., 2018; Rubalcaba et al., 2020; Verberk et al., 2011).

In contrast to the lower-latitude populations, among large individuals from the higher-latitude population with a lower mean temperature and higher thermal variability, the mass-specific SMR continued to increase (*E*=0.89 eV) at temperatures above the current maxima. This implies that individuals across all mass classes of the higher-latitude population did not reach their thermal limits at the forecasted temperatures. In line with our observation, Rohr et al. (2018) observed that thermal tolerance increases with latitude. Physiological plasticity is often proportional to the degree of variation in local temperature that a species experiences in its original habitat: populations inhabiting more variable thermal environments are expected to have broader thermal tolerance (Rohr et al., 2018) and higher plasticity (Sun et al., 2022) than those inhabiting more temperate environments (but see Seebacher et al., 2015). This implies that populations from lower latitudes with more constant climates are more sensitive to temperature abnormalities because they also live closer to their thermal and physiological limits (Sunday et al., 2011; Thyrring et al., 2020).

In this regard, our findings show that the larger conspecifics living at lower latitudes, owing to either the limitation of the performance of metabolic enzymes or the availability of oxygen, could be the first to experience the negative impacts of future warming on performance and other metabolism-related processes. This adds to growing evidence that physiological constraints at warmer temperatures reduce the performance of large-bodied individuals to a greater degree than their small-bodied conspecifics (Lindmark et al., 2022). This in turn affects population size structure in the face of climate change, particularly in populations living at the edge of their physiological tolerance (Huss et al., 2019; Neuheimer et al., 2011).

It is important to bear in mind that although temperature manipulation experiments to test and develop the relevant theoretical frameworks and climate-based models are clearly needed, uncertainties that might affect the interpretation of the findings remain. The main limitation of our study was that the thermal acclimation period (here serving merely to minimize stress and thermal shock) was much shorter than the acclimatization of organisms in the face of climate change. In nature, animals may have a far longer period to cope with predicted climate change and may offset this effect by means of either phenotype plasticity or genotype evolution. In addition, in order to maintain functionality, ectothermic animals may seek refuge or migrate to a cooler place (behavioural buffering) as ambient water temperatures begin to exceed their upper thermal tolerance limits. Long-term experimental studies of metabolic rates involving the manipulation of climate change scenarios are an important research priority as part of efforts to develop our understanding of underlying mechanisms in the face of climate change.

In conclusion, in this study, we provide further evidence that a marginal temperature increase of 1.2°C under future climate change scenarios impacts the SMR of individuals and populations, and thus even the most conservative IPCC forecast seems likely to affect ecosystem functioning. Given the evidence for interactive temperature–mass effects on metabolic rate and their dependence on latitude, caution must be exercised, especially when predicting population responses to climate change, which have profound implications for all metabolism-related processes.

## Supplementary Material

10.1242/jexbio.244842_sup1 Supplementary information

## References

[JEB244842C1] Able, K. W. and Hagan, S. M. (2000). Effects of common reed (*Phragmites australis*) invasion on marsh surface macrofauna: response of fishes and decapod crustaceans. *Estuaries* 23, 633-646. 10.2307/1352890

[JEB244842C2] Angilletta, M. J. (2009). *Thermal Adaptation: A Theoretical and Empirical Synthesis*. Oxford: Oxford University Press. 10.1093/acprof:oso/9780198570875.001.1

[JEB244842C3] Anttila, K., Dhillon, R. S., Boulding, E. G., Farrell, A. P., Glebe, B. D., Elliott, J. A. K., Wolters, W. R. and Schulte, P. M. (2013). Variation in temperature tolerance among families of Atlantic salmon (Salmo salar) is associated with hypoxia tolerance, ventricle size and myoglobin level. *J. Exp. Biol.* 216, 1183-1190. 10.1242/jeb.080556 23487268

[JEB244842C4] Arrhenius, S. (1889). Uber die reaktionsgeschwindigkeit bei der inversion von rohrzucker durcj sauren. *Z. Phys. Chem.* 4, 226-248. 10.1515/zpch-1889-0416

[JEB244842C5] Atkinson, D., Morley, S. A. and Hughes, R. N. (2006). From cells to colonies: at what levels of body organization does the ‘temperature-size rule’ apply? *Evol. Dev.* 8, 202-214. 10.1111/j.1525-142X.2006.00090.x 16509898

[JEB244842C6] Audzijonyte, A., Richards, S. A., Stuart-Smith, R. D., Pecl, G., Edgar, G. J., Barrett, N. S., Payne, N. and Blanchard, J. L. (2020). Fish body sizes change with temperature but not all species shrink with warming. *Nat. Ecol. Evol.* 4, 809-814. 10.1038/s41559-020-1171-0 32251381

[JEB244842C7] Baltazar-Soares, M., Paiva, F., Chen, Y., Zhan, A. and Briski, E. (2017). Diversity and distribution of genetic variation in gammarids: Comparing patterns between invasive and non-invasive species. *Ecol. Evol.* 7, 7687-7698. 10.1002/ece3.3208 29043025 PMC5632605

[JEB244842C8] Barria, A. M., Bacigalupe, L. D., Lagos, N. A. and Lardies, M. A. (2018). Thermal physiological traits and plasticity of metabolism are sensitive to biogeographic breaks in a rock-pool marine shrimp. *J. Exp. Biol.* 221, jeb181008. 10.1242/jeb.181008 30072385

[JEB244842C9] Bates, D., Mächler, M., Bolker, B. and Walker, S. (2015). Fitting linear mixed-effects models using lme4. *J. Stat. Softw.* 67, 1-48. 10.18637/jss.v067.i01

[JEB244842C10] Bayne, B. L. (2017). *Biology of Oysters*. London: Elsevier Academic Press.

[JEB244842C11] Bellan-Santini, D., Diviacco, G., Krapp-Schickel, G. and Ruffo, S. (1989). *The Amphipoda of the Mediterranean 2, Gammaridea (Haustoriidae to Lysianassidae)*. Monaco: Musée Océanographique.

[JEB244842C12] Bellan-Santini, D., Karaman, G. S., Ledoyer, M., Myers, A., Ruffo, S. and Vader, W. (1998). *The Amphipoda of the Mediterranean. Part 4: Localities and Map, Addenda to Parts 1-3, Key to Families, Ecology, Faunistics and Zoogeography*. Monaco: Musée Océanographique.

[JEB244842C13] Benavente, J. N., Fryxell, D. C., Kinnison, M. T., Palkovacs, E. P. and Simon, K. S. (2022). Plasticity and evolution shape the scaling of metabolism and excretion along a geothermal temperature gradient. *Funct. Ecol.* 36, 1303-1314. 10.1111/1365-2435.14020

[JEB244842C14] Bennett, S., Duarte, C. M., Marbà, N. and Wernberg, T. (2019). Integrating within-species variation in thermal physiology into climate change ecology. *Philos. Trans. R. Soc. B Biol. Sci.* 374, 20180550. 10.1098/rstb.2018.0550 PMC6606463 31203756

[JEB244842C15] Bestion, E., Teyssier, A., Richard, M., Clobert, J. and Cote, J. (2015). Live fast, die young: experimental evidence of population extinction risk due to climate change. *PLoS Biol.* 13, e1002281. 10.1371/journal.pbio.1002281 26501958 PMC4621050

[JEB244842C16] Boardman, L. and Terblanche, J. S. (2015). Oxygen safety margins set thermal limits in an insect model system. *J. Exp. Biol.* 218, 1677-1685. 10.1242/jeb.120261 26041031

[JEB244842C17] Brandl, S. J., Lefcheck, J. S., Bates, A. E., Rasher, D. B. and Norin, T. (2022). Can metabolic traits explain animal community assembly and functioning? *Biol. Rev.* 111*,* 549*.* 10.1111/brv.12892 36054431

[JEB244842C18] Brown, J. H., Gillooly, J. F., Allen, A. P., Savage, V. M. and West, G. B. (2004). Toward a metabolic theory of ecology. *Ecology* 85, 1771-1789. 10.1890/03-9000

[JEB244842C19] Bruno, J. F., Carr, L. A. and O'Connor, M. I. (2015). Exploring the role of temperature in the ocean through metabolic scaling. *Ecology* 96, 3126-3140. 10.1890/14-1954.1 26909420

[JEB244842C20] Bullock, T. H. (1955). Compensation for temperature in the metabolism activity of poikilotherms. *Biol. Rev.* 30, 311-342. 10.1111/j.1469-185X.1955.tb01211.x

[JEB244842C21] Buongiorno Nardelli, B., Tronconi, C., Pisano, A. and Santoleri, R. (2013). High and ultra-high resolution processing of satellite sea surface temperature data over Southern European seas in the framework of MyOcean project. *Remote Sens. Environ.* 129, 1-16. 10.1016/j.rse.2012.10.012

[JEB244842C22] Burton, T., Killen, S. S., Armstrong, J. D. and Metcalfe, N. B. (2011). What causes intraspecific variation in resting metabolic rate and what are its ecological consequences? *Proc. R. Soc. B Biol. Sci.* 278, 3465-3473. 10.1098/rspb.2011.1778 PMC3189380 21957133

[JEB244842C23] Careau, V., Thomas, D., Humphries, M. M. and Réale, D. (2008). Energy metabolism and animal personality. *Oikos* 117, 641-653. 10.1111/j.0030-1299.2008.16513.x

[JEB244842C24] Carey, N. and Sigwart, J. D. (2014). Size matters: plasticity in metabolic scaling shows body-size may modulate responses to climate change. *Biol. Lett.* 10, 20140408. 10.1098/rsbl.2014.0408 25122741 PMC4155909

[JEB244842C25] Chown, S. and Gaston, K. (1999). Exploring links between physiology and ecology at macro-scales: the role of respiratory metabolism in insects. *Biol. Rev.* 74, 87-120. 10.1017/S000632319800526X

[JEB244842C26] Chown, S. L., van der Merwe, M. and Smith, V. R. (1997). The influence of habitat and altitude on oxygen uptake in sub-Antarctic weevils. *Physiol. Zool.* 70, 116-124. 10.1086/639554 9231383

[JEB244842C27] Clarke, A. (1991). What is cold adaptation and how should we measure it? *Amer. Zool.* 31, 81-92. 10.1093/icb/31.1.81

[JEB244842C28] Clarke, A. (1993). Seasonal acclimatization and latitudinal compensation in metabolism: do they exist? *Funct. Ecol.* 7, 139-149. 10.2307/2389880

[JEB244842C29] Clarke, A. and Fraser, K. P. P. (2004). Why does metabolism scale with temperature? *Funct. Ecol.* 18, 243-251. 10.1111/j.0269-8463.2004.00841.x

[JEB244842C30] %CMEMS (Copernicus Marine Environment Monitoring Service) (2011). Mediterranean Sea - high resolution and ultra high resolution L3S sea surface temperature. 10.48670/moi-00171

[JEB244842C31] Coggins, B. L., Anderson, C. E., Hasan, R., Pearson, A. C., Ekwudo, M. N., Bidwell, J. R. and Yampolsky, L. Y. (2021). Breaking free from thermodynamic constraints: thermal acclimation and metabolic compensation in a freshwater zooplankton species. *J. Exp. Biol.* 224, jeb237727. 10.1242/jeb.237727 33328286

[JEB244842C32] Costello, M. and Emblow, J. (2001). *European Register of Marine Species: a Check-List of the Marine Species in Europe and a Bibliography of Guides to Their Identification*. Paris: Muséum national d'Histoire naturelle, Paris, Collection Patrimoines Naturels.

[JEB244842C33] Cozzoli, F., Shokri, M., Ligetta, G., Ciotti, M., Gjoni, V., Marrocco, V., Vignes, F. and Basset, A. (2020). Relationship between individual metabolic rate and patch departure behaviour: evidence from aquatic gastropods. *Oikos* 129, 1657-1667. 10.1111/oik.07378

[JEB244842C34] Cozzoli, F., Shokri, M., Gomes da Conceição, T., Herman, P. M. J., Hu, Z., Soissons, L. M., Van Dalen, J., Ysebaert, T. and Bouma, T. J. (2021). Modelling spatial and temporal patterns in bioturbator effects on sediment resuspension: abiophysical metabolic approach. *Sci. Total Environ.* 792, 148215. 10.1016/j.scitotenv.2021.148215 34465034

[JEB244842C35] Cozzoli, F., Shokri, M., Boulamail, S., Marrocco, V., Vignes, F. and Basset, A. (2022). The size dependency of foraging behaviour: an empirical test performed on aquatic amphipods. *Oecologia* 199, 377-386. 10.1007/s00442-022-05195-8 35678931 PMC9225974

[JEB244842C36] Daufresne, M., Lengfellner, K. and Sommer, U. (2009). Global warming benefits the small in aquatic ecosystems. *Proc. Natl. Acad. Sci. USA* 106, 12788-12793. 10.1073/pnas.0902080106 19620720 PMC2722360

[JEB244842C37] DeLong, J. P., Gibert, J. P., Luhring, T. M., Bachman, G., Reed, B., Neyer, A. and Montooth, K. L. (2017). The combined effects of reactant kinetics and enzyme stability explain the temperature dependence of metabolic rates. *Ecol. Evol.* 7, 3940-3950. 10.1002/ece3.2955 28616189 PMC5468145

[JEB244842C38] Fangue, N. A., Richards, J. G. and Schulte, P. M. (2009). Do mitochondrial properties explain intraspecific variation in thermal tolerance? *J. Exp. Biol.* 212, 514-522. 10.1242/jeb.024034 19181899

[JEB244842C39] Forster, J., Hirst, A. G. and Atkinson, D. (2012). Warming-induced reductions in body size are greater in aquatic than terrestrial species. *Proc. Natl. Acad. Sci. USA* 109, 19310-19314. 10.1073/pnas.1210460109 23129645 PMC3511100

[JEB244842C40] Fossen, E. I. F., Pélabon, C. and Einum, S. (2019). Genetic and environmental effects on the scaling of metabolic rate with body size. *J. Exp. Biol.* 222, jeb193243. 10.1242/jeb.193243 30910836

[JEB244842C41] Gardner, J. L., Peters, A., Kearney, M. R., Joseph, L. and Heinsohn, R. (2011). Declining body size: a third universal response to warming? *Trends Ecol. Evol.* 26, 285-291. 10.1016/j.tree.2011.03.005 21470708

[JEB244842C42] Gaston, K. J., Blackburn, T. M. and Spicer, J. I. (1998). Rapoport's rule: time for an epitaph? *Trends Ecol. Evol.* 13, 70-74. 10.1016/S0169-5347(97)01236-6 21238203

[JEB244842C43] Genner, M. J., Freer, J. and A. Rutterford, L. (2017). *Future of the Sea: Biological Responses to Ocean Warming*. London: Foresight, Government Office for Science.

[JEB244842C44] Gerhardt, A., Bloor, M. and Lloyd, M. C. (2011). Gammarus: important taxon in freshwater and marine changing environments. *Int. J. Zool.* 2011, 1-2. 10.1155/2011/524276

[JEB244842C45] Gillooly, J. F., Brown, J. H., West, G. B., Savage, V. M. and Charnov, E. L. (2001). Effects of size and temperature on metabolic rate. *Science* 293, 2248-2251. 10.1126/science.1061967 11567137

[JEB244842C46] Gjoni, V., Basset, A. and Glazier, D. S. (2020). Temperature and predator cues interactively affect ontogenetic metabolic scaling of aquatic amphipods. *Biol. Lett.* 16, 20200267. 10.1098/rsbl.2020.0267 32673549 PMC7423044

[JEB244842C47] Glazier, D. S. (2005). Beyond the ‘3/4-power law’: variation in the intra- and interspecific scaling of metabolic rate in animals. *Biol. Rev.* 80, 611-662. 10.1017/S1464793105006834 16221332

[JEB244842C48] Glazier, D. S. (2014). Scaling of metabolic scaling within physical limits. *Systems* 2, 425-450. 10.3390/systems2040425

[JEB244842C49] Glazier, D. S. (2015). Is metabolic rate a universal ‘pacemaker’ for biological processes? *Biol. Rev.* 90, 377-407. 10.1111/brv.12115 24863680

[JEB244842C50] Glazier, D. S. (2020). Activity alters how temperature influences intraspecific metabolic scaling: testing the metabolic-level boundaries hypothesis. *J. Comp. Physiol. B.* 190, 445-454. 10.1007/s00360-020-01279-0 32388580

[JEB244842C51] Glazier, D. S. (2021). Biological scaling analyses are more than statistical line fitting. *J. Exp. Biol.* 224, jeb241059. 10.1242/jeb.241059 34086905

[JEB244842C52] Glazier, D. S. (2022). How metabolic rate relates to cell size. *Biology* 11, 1106. 10.3390/biology11081106 35892962 PMC9332559

[JEB244842C53] Glazier, D. S. and Sparks, B. (1997). Energetics of amphipods in ion-poor waters: stress resistance is not invariably linked to low metabolic rates. *Funct. Ecol.* 11, 126-128. 10.1046/j.1365-2435.1997.00061.x

[JEB244842C54] Glazier, D. S., Butler, E. M., Lombardi, S. A., Deptola, T. J., Reese, A. J. and Satterthwaite, E. V. (2011). Ecological effects on metabolic scaling: amphipod responses to fish predators in freshwater springs. *Ecol. Monogr.* 81, 599-618. 10.1890/11-0264.1

[JEB244842C55] Glazier, D. S., Gring, J. P., Holsopple, J. R. and Gjoni, V. (2020). Temperature effects on metabolic scaling of a keystone freshwater crustacean depend on fish-predation regime. *J. Exp. Biol.* 223, jeb232322. 10.1242/jeb.232322 33037112

[JEB244842C56] Gnaiger, E. (1983). *Calculation of Energetic and Biochemical Equivalents of Respiratory Oxygen Consumption.* Berlin: Springer.

[JEB244842C57] Groemping, U. (2006). Relative importance for linear regression in R: the package relaimpo. *J. Stat. Softw.* 17, 1-27. 10.1360/jos170001

[JEB244842C58] Grolemund, G. and Wickham, H. (2011). Dates and times made easy with Lubridate. *J. Stat. Softw.* 40, 1-25. 10.18637/jss.v040.i03

[JEB244842C59] Hickling, R., Roy, D. B., Hill, J. K., Fox, R. and Thomas, C. D. (2006). The distributions of a wide range of taxonomic groups are expanding polewards. *Glob. Chang. Biol.* 12, 450-455. 10.1111/j.1365-2486.2006.01116.x

[JEB244842C60] Hochachka, P. W. and Somero, G. N. (2002). *Biochemical Adaptation: Mechanism and Process in Physiological Evolution*. New York: Oxford University Press.

[JEB244842C61] Hoefnagel, K. N. and Verberk, W. C. E. P. (2015). Is the temperature-size rule mediated by oxygen in aquatic ectotherms? *J. Therm. Biol.* 54, 56-65. 10.1016/j.jtherbio.2014.12.003 26615727

[JEB244842C62] Hoffmann, A. A., Shirriffs, J. and Scott, M. (2005). Relative importance of plastic vs genetic factors in adaptive differentiation: geographical variation for stress resistance in *Drosophila melanogaster* from eastern Australia. *Funct. Ecol.* 19, 222-227. 10.1111/j.1365-2435.2005.00959.x

[JEB244842C63] Huss, M., Lindmark, M., Jacobson, P., van Dorst, R. M. and Gårdmark, A. (2019). Experimental evidence of gradual size-dependent shifts in body size and growth of fish in response to warming. *Glob. Chang. Biol.* 25, 2285-2295. 10.1111/gcb.14637 30932292 PMC6850025

[JEB244842C64] IPCC (2014). Climate change 2014: synthesis report. In *Contribution of Working Groups I, II and III to the Fifth Assessment Report of the Iintergovernmental Panel on Climate Change* (ed. Core Writing Team, R. K. Pachauri and L. A. Meyer). Geneva: IPCC.

[JEB244842C65] James, F. C. (1970). Geographic size variation in birds and its relationship to climate. *Ecology* 51, 365-390. 10.2307/1935374

[JEB244842C66] Job, S. V. (1957). The routine-active oxygen consumption of the milk fish. *Proc. Indian Acad. Sci.* 45, 302-313. 10.1007/BF03051032

[JEB244842C67] Jutfelt, F., Norin, T., Ern, R., Overgaard, J., Wang, T., McKenzie, D. J., Lefevre, S., Nilsson, G. E., Metcalfe, N. B., Hickey, A. J. R. et al. (2018). Oxygen- and capacity-limited thermal tolerance: blurring ecology and physiology. *J. Exp. Biol.* 221, jeb169615. 10.1242/jeb.169615 29321291

[JEB244842C68] Kefford, B. J., Ghalambor, C. K., Dewenter, B., Poff, N. L., Hughes, J., Reich, J., Thompson, R. (2022). Acute, diel, and annual temperature variability and the thermal biology of ectotherms. *Glob. Chang. Biol*. 28, 6872-6888. 10.1111/gcb.16453 36177681 PMC9828456

[JEB244842C69] Killen, S. S., Atkinson, D. and Glazier, D. S. (2010). The intraspecific scaling of metabolic rate with body mass in fishes depends on lifestyle and temperature. *Ecol. Lett.* 13, 184-193. 10.1111/j.1461-0248.2009.01415.x 20059525

[JEB244842C70] Killen, S. S., Glazier, D. S., Rezende, E. L., Clark, T. D., Atkinson, D., Willener, A. S. T. and Halsey, L. G. (2016). Ecological influences and morphological correlates of resting and maximal metabolic rates across teleost fish species. *Am. Nat.* 187, 592-606. 10.1086/685893 27104992

[JEB244842C71] Kleiber, M. (1932). Body size and metabolism. *Hilgardia* 6, 315-353. 10.3733/hilg.v06n11p315

[JEB244842C72] Kordas, R. L., Pawar, S., Kontopoulos, D.-G., Woodward, G. and O'Gorman, E. J. (2022). Metabolic plasticity can amplify ecosystem responses to global warming. *Nat. Commun.* 13, 639. 10.1038/s41467-022-29808-1 35443761 PMC9021271

[JEB244842C73] Kraemer, B. M., Chandra, S., Dell, A. I., Dix, M., Kuusisto, E., Livingstone, D. M., Schladow, S. G., Silow, E., Sitoki, L. M., Tamatamah, R. et al. (2017). Global patterns in lake ecosystem responses to warming based on the temperature dependence of metabolism. *Glob. Chang. Biol.* 23, 1881-1890. 10.1111/gcb.13459 27591144

[JEB244842C74] Leiva, F. P., Garcés, C., Verberk, W. C. E. P., Care, M., Paschke, K. and Gebauer, P. (2018). Differences in the respiratory response to temperature and hypoxia across four life-stages of the intertidal porcelain crab *Petrolisthes laevigatus*. *Mar. Biol.* 165, 146. 10.1007/s00227-018-3406-z 30220736 PMC6132507

[JEB244842C75] Leiva, F. P., Calosi, P. and Verberk, W. C. E. P. (2019). Scaling of thermal tolerance with body mass and genome size in ectotherms: a comparison between water- and air-breathers. *Philos. Trans. R. Soc. Lond. B. Biol. Sci.* 374, 20190035. 10.1098/rstb.2019.0035 31203753 PMC6606457

[JEB244842C76] Lindeman, R., Merenda, P. and Gold, R. (1980). *Introduction to Bivariate and Multivariate Analysis*. Glenview, IL: Scott, Foresman.

[JEB244842C77] Lindmark, M., Huss, M., Ohlberger, J. and Gårdmark, A. (2018). Temperature-dependent body size effects determine population responses to climate warming. *Ecol. Lett.* 21, 181-189. 10.1111/ele.12880 29161762

[JEB244842C78] Lindmark, M., Ohlberger, J. and Gårdmark, A. (2022). Optimum growth temperature declines with body size within fish species. *Glob. Chang. Biol.* 28, 2259-2271. 10.1111/gcb.16067 35060649

[JEB244842C79] Magozzi, S. and Calosi, P. (2015). Integrating metabolic performance, thermal tolerance, and plasticity enables for more accurate predictions on species vulnerability to acute and chronic effects of global warming. *Glob. Chang. Biol.* 21, 181-194. 10.1111/gcb.12695 25155644

[JEB244842C80] Nati, J. J. H., Svendsen, M. B. S., Marras, S., Killen, S. S., Steffensen, J. F., McKenzie, D. J. and Domenici, P. (2021). Intraspecific variation in thermal tolerance differs between tropical and temperate fishes. *Sci. Rep.* 11, 21272. 10.1038/s41598-021-00695-8 34711864 PMC8553816

[JEB244842C81] Naya, D. E., Naya, H. and White, C. R. (2018). On the interplay among ambient temperature, basal metabolic rate, and body mass. *Am. Nat.* 192, 518-524. 10.1086/698372 30205024

[JEB244842C82] Nelson, D. (2011). *Gammarus*–microbial interactions: a review. *Int. J. Zool.* 2011, 1-6. 10.1155/2011/295026

[JEB244842C83] Neuheimer, A. B., Thresher, R. E., Lyle, J. M. and Semmens, J. M. (2011). Tolerance limit for fish growth exceeded by warming waters. *Nat. Clim. Chang.* 1, 110-113. 10.1038/nclimate1084

[JEB244842C84] O'Connor, M. I., Piehler, M. F., Leech, D. M., Anton, A. and Bruno, J. F. (2009). Warming and resource availability shift food web structure and metabolism. *PLoS Biol.* 7, e1000178. 10.1371/journal.pbio.1000178 19707271 PMC2723928

[JEB244842C85] Ohlberger, J., Mehner, T., Staaks, G. and Hölker, F. (2012). Intraspecific temperature dependence of the scaling of metabolic rate with body mass in fishes and its ecological implications. *Oikos* 121, 245-251. 10.1111/j.1600-0706.2011.19882.x

[JEB244842C86] Parmesan, C. and Yohe, G. (2003). A globally coherent fingerprint of climate change impacts across natural systems. *Nature* 421, 37-42. 10.1038/nature01286 12511946

[JEB244842C87] Pauly, D. (2010). *Gasping Fish and Panting Squids: Oxygen, Temperature and the Growth of Water-Breathing Animals*. Oldendorf, Luhe: International Ecology Institute.

[JEB244842C88] Peralta-Maraver, I. and Rezende, E. L. (2021). Heat tolerance in ectotherms scales predictably with body size. *Nat. Clim. Chang.* 11, 58-63. 10.1038/s41558-020-00938-y

[JEB244842C89] Poloczanska, E. S., Brown, C. J., Sydeman, W. J., Kiessling, W., Schoeman, D. S., Moore, P. J., Brander, K., Bruno, J. F., Buckley, L. B., Burrows, M. T. et al. (2013). Global imprint of climate change on marine life. *Nat. Clim. Chang.* 3, 919-925. 10.1038/nclimate1958

[JEB244842C90] Pörtner, H. O. and Gutt, J. (2016). Impacts of climate variability and change on (marine) animals: physiological underpinnings and evolutionary consequences. *Integr. Comp. Biol.* 56, 31-44. 10.1093/icb/icw019 27371560

[JEB244842C91] Pörtner, H. O. and Knust, R. (2007). Climate change affects marine fishes through the oxygen limitation of thermal tolerance. *Science* 315, 95-97. 10.1126/science.1135471 17204649

[JEB244842C92] Precht, H., Laudien, H. and Havsteen, B. (1973). The normal temperature range. In *Temperature and Life* (ed. H. Precht, J. Christophersen, H. Hensel and W. Larcher), pp. 302-399. New York: Springer.

[JEB244842C93] Rao, K. P. and Bullock, T. H. (1954). Q_10_ as a function of size and habitat temperature in poikilotherms. *Am. Nat.* 88, 33-44. 10.1086/281806

[JEB244842C94] Rohr, J. R., Civitello, D. J., Cohen, J. M., Roznik, E. A., Sinervo, B. and Dell, A. I. (2018). The complex drivers of thermal acclimation and breadth in ectotherms. *Ecol. Lett.* 21, 1425-1439. 10.1111/ele.13107 30009486

[JEB244842C95] Rubalcaba, J. G., Verberk, W. C. E. P., Hendriks, A. J., Saris, B. and Woods, H. A. (2020). Oxygen limitation may affect the temperature and size dependence of metabolism in aquatic ectotherms. *Proc. Natl. Acad. Sci. USA* 117, 31963-31968. 10.1073/pnas.2003292117 33257544 PMC7749359

[JEB244842C96] Santini, L., Cornulier, T., Bullock, J. M., Palmer, S. C. F., White, S. M., Hodgson, J. A., Bocedi, G. and Travis, J. M. J. (2016). A trait-based approach for predicting species responses to environmental change from sparse data: how well might terrestrial mammals track climate change? *Glob. Chang. Biol.* 22, 2415-2424. 10.1111/gcb.13271 27073017

[JEB244842C97] Schulte, P. M. (2015). The effects of temperature on aerobic metabolism: towards a mechanistic understanding of the responses of ectotherms to a changing environment. *J. Exp. Biol.* 218, 1856-1866. 10.1242/jeb.118851 26085663

[JEB244842C98] Schulte, P. M., Healy, T. M. and Fangue, N. A. (2011). Thermal performance curves, phenotypic plasticity, and the time scales of temperature exposure. *Integr. Comp. Biol.* 51, 691-702. 10.1093/icb/icr097 21841184

[JEB244842C99] Schwartzkopff, J. (1955). Vergleichende untersuchungen der hertzfrequenz bei krebsen. *Biol. Zentr.* 74, 97-480.

[JEB244842C100] Seebacher, F., White, C. R. and Franklin, C. E. (2015). Physiological plasticity increases resilience of ectothermic animals to climate change. *Nat. Clim. Chang.* 5, 61-66. 10.1038/nclimate2457

[JEB244842C101] Seibel, B. A., Dymowska, A. and Rosenthal, J. (2007). Metabolic temperature compensation and coevolution of locomotory performance in pteropod molluscs. *Integr. Comp. Biol.* 47, 880-891. 10.1093/icb/icm089 21669767

[JEB244842C102] Semsar-kazerouni, M. and Verberk, W. C. E. P. (2018). It's about time: linkages between heat tolerance, thermal acclimation and metabolic rate at different temporal scales in the freshwater amphipod *Gammarus fossarum* Koch, 1836. *J. Therm. Biol.* 75, 31-37. 10.1016/j.jtherbio.2018.04.016 30017049

[JEB244842C103] Shadrin, N., Yakovenko, V. and Anufriieva, E. (2022). Feeding behavior of *Gammarus aequicauda* in the presence of two prey species of *Artemia* sp. and *Baeotendipes noctivagus*. *J. Exp. Zool. A Ecol. Integr. Physiol.* 337, 768-775. 10.1002/jez.2635 35713191

[JEB244842C104] Shokri, M., Ciotti, M., Vignes, F., Gjoni, V. and Basset, A. (2019). Components of standard metabolic rate variability in three species of gammarids. *Web Ecol.* 19, 1-13. 10.5194/we-19-1-2019

[JEB244842C105] Shokri, M., Cozzoli, F., Ciotti, M., Gjoni, V., Marrocco, V., Vignes, F. and Basset, A. (2021). A new approach to assessing the space use behavior of macroinvertebrates by automated video tracking. *Ecol. Evol.* 11, 3004-3014. 10.1002/ece3.7129 33841762 PMC8019041

[JEB244842C106] Silva-Garay, L. and Lowe, C. G. (2021). Effects of temperature and body-mass on the standard metabolic rates of the round stingray, *Urobatis halleri* (Cooper, 1863). *J. Exp. Mar. Bio. Ecol.* 540, 151564. 10.1016/j.jembe.2021.151564

[JEB244842C107] Sinclair, B. J., Marshall, K. E., Sewell, M. A., Levesque, D. L., Willett, C. S., Slotsbo, S., Dong, Y., Harley, C. D. G., Marshall, D. J., Helmuth, B. S. et al. (2016). Can we predict ectotherm responses to climate change using thermal performance curves and body temperatures? *Ecol. Lett.* 19, 1372-1385. 10.1111/ele.12686 27667778

[JEB244842C108] Sokolova, I. M. and Pörtner, H. O. (2003). Metabolic plasticity and critical temperatures for aerobic scope in a eurythermal marine invertebrate (*Littorina saxatilis*, Gastropoda: Littorinidae) from different latitudes. *J. Exp. Biol.* 206, 195-207. 10.1242/jeb.00054 12456709

[JEB244842C109] Stock, J. H. (1966). A key to the species of the locusta-group of the amphipod genus *Gammarus*, with notes on their nomenclature. *Bull. Zool. Museum* 1, 1-5.

[JEB244842C110] Sun, B., Williams, C. M., Li, T., Speakman, J. R., Jin, Z., Lu, H., Luo, L. and Du, W. (2022). Higher metabolic plasticity in temperate compared to tropical lizards suggests increased resilience to climate change. *Ecol. Monogr.* 92, e1512.

[JEB244842C111] Sunday, J. M., Bates, A. E. and Dulvy, N. K. (2011). Global analysis of thermal tolerance and latitude in ectotherms. *Proc. R. Soc. B Biol. Sci.* 278, 1823-1830. 10.1098/rspb.2010.1295 PMC3097822 21106582

[JEB244842C112] Sunday, J., Bennett, J. M., Calosi, P., Clusella-Trullas, S., Gravel, S., Hargreaves, A. L., Leiva, F. P., Verberk, W. C. E. P., Olalla-Tárraga, M. Á. and Morales-Castilla, I. (2019). Thermal tolerance patterns across latitude and elevation. *Philos. Trans. R. Soc. B Biol. Sci.* 374, 20190036. 10.1098/rstb.2019.0036 PMC6606462 31203755

[JEB244842C113] Terblanche, J. S. and Chown, S. L. (2006). The relative contributions of developmental plasticity and adult acclimation to physiological variation in the tsetse fly, *Glossina pallidipes* (Diptera, Glossinidae). *J. Exp. Biol.* 209, 1064-1073. 10.1242/jeb.02129 16513933 PMC1431687

[JEB244842C114] Terblanche, J. S., Clusella–Trullas, S., Deere, J. A., Van Vuuren, B. J. and Chown, S. L. (2009). Directional evolution of the slope of the metabolic rate–temperature relationship is correlated with climate. *Physiol. Biochem. Zool.* 82, 495-503. 10.1086/605361 19624273

[JEB244842C115] Thyrring, J., Tremblay, R. and Sejr, M. K. (2020). Local cold adaption increases the thermal window of temperate mussels in the Arctic. *Conserv. Physiol.* 7, 1-10. 10.1093/conphys/coz098 PMC6933310 31890211

[JEB244842C116] Tillin, H. and White, N. (2017). Marine life information network: biology and sensitivity key information reviews. Plymouth: Marine Biological Association of the United Kingdom. https://www.marlin.ac.uk/species/detail/1142.

[JEB244842C117] Vasseur, D. A., DeLong, J. P., Gilbert, B., Greig, H. S., Harley, C. D. G., McCann, K. S., Savage, V., Tunney, T. D. and O'Connor, M. I. (2014). Increased temperature variation poses a greater risk to species than climate warming. *Proc. R. Soc. B Biol. Sci.* 281, 20132612. 10.1098/rspb.2013.2612 PMC3924069 24478296

[JEB244842C118] Verberk, W. C. E. P. and Atkinson, D. (2013). Why polar gigantism and Palaeozoic gigantism are not equivalent: effects of oxygen and temperature on the body size of ectotherms. *Funct. Ecol.* 27, 1275-1285. 10.1111/1365-2435.12152

[JEB244842C119] Verberk, W. C., Bilton, D. T., Calosi, P. and Spicer, J. I. (2011). Oxygen supply in aquatic ectotherms: partial pressure and solubility together explain biodiversity and size patterns. *Ecology* 92, 1565-1572. 10.1890/10-2369.1 21905423

[JEB244842C120] Verberk, W. C. E. P., Overgaard, J., Ern, R., Bayley, M., Wang, T., Boardman, L. and Terblanche, J. S. (2016a). Does oxygen limit thermal tolerance in arthropods? A critical review of current evidence. *Comp. Biochem. Physiol. Part A Mol. Integr. Physiol.* 192, 64-78. 10.1016/j.cbpa.2015.10.020 PMC4717866 26506130

[JEB244842C121] Verberk, W. C. E. P., Bartolini, F., Marshall, D. J., Pörtner, H. O., Terblanche, J. S., White, C. R. and Giomi, F. (2016b). Can respiratory physiology predict thermal niches? *Ann. N. Y. Acad. Sci.* 1365, 73-88. 10.1111/nyas.12876 26333058

[JEB244842C122] Verberk, W. C. E. P., Buchwalter, D. B. and Kefford, B. J. (2020). Energetics as a lens to understanding aquatic insect's responses to changing temperature, dissolved oxygen and salinity regimes. *Curr. Opin. Insect Sci.* 41, 46-53. 10.1016/j.cois.2020.06.001 32682316

[JEB244842C123] Vernberg, J. (1959). Studies on the physiological variation between tropical and temperate zone fiddler crabs of the genus *Uca*. III. the influence of temperature acclimation on oxygen consumption of whole organisms. *Biol. Bull.* 117, 582-593. 10.2307/1538868

[JEB244842C124] Węsławski, J. M., Legeżyńska, J. and Włodarska-Kowalczuk, M. (2020). Will shrinking body size and increasing species diversity of crustaceans follow the warming of the Arctic littoral? *Ecol. Evol.* 10, 10305-10313. 10.1002/ece3.6780 33072260 PMC7548195

[JEB244842C125] West, G. B., Brown, J. H. and Enquist, B. J. (1997). A general model for the origin of allometric scaling laws in biology. *Science* 276, 122-126. 10.1126/science.276.5309.122 9082983

[JEB244842C126] White, C. R., Alton, L. A. and Frappell, P. B. (2012). Metabolic cold adaptation in fishes occurs at the level of whole animal, mitochondria and enzyme. *Proc. R. Soc. B Biol. Sci.* 279, 1740-1747. 10.1098/rspb.2011.2060 PMC3297453 22158960

[JEB244842C127] Wickham, H., Averick, M., Bryan, J., Chang, W., McGowan, L. D., François, R., Grolemund, G., Hayes, A., Henry, L. and Hester, J. (2019). Welcome to the Tidyverse. *J. Open Source Softw.* 4, 1686. 10.21105/joss.01686

[JEB244842C128] Wrona, F. J. and Davies, R. W. (1984). An improved flow-through respirometer for aquatic macroinvertebrate bioenergetic research. *Can. J. Fish. Aquat. Sci.* 41, 380-385. 10.1139/f84-042

[JEB244842C129] Young, S. R. (1979). Effect of temperature change on the metabolic rate of an Antarctic mite. *J. Comp. Physiol.* 131, 341-346. 10.1007/BF00688809

[JEB244842C130] Yvon-Durocher, G., Jones, J. I., Trimmer, M., Woodward, G. and Montoya, J. M. (2010). Warming alters the metabolic balance of ecosystems. *Philos. Trans. R. Soc. Lond. B Biol. Sci.* 365, 2117-2126. 10.1098/rstb.2010.0038 20513719 PMC2880133

