# Supplementary material for: Metabolic rate and climate change across latitudes: evidence of mass-dependent responses in aquatic amphipods
Source: J Exp Biol. 2022 Nov 25;225(22):jeb244842. doi: 10.1242/jeb.244842 (PMC9720750; doi:10.1242/jeb.244842)
Supplement: Supplementary information [file jexbio-225-244842-s1.pdf]

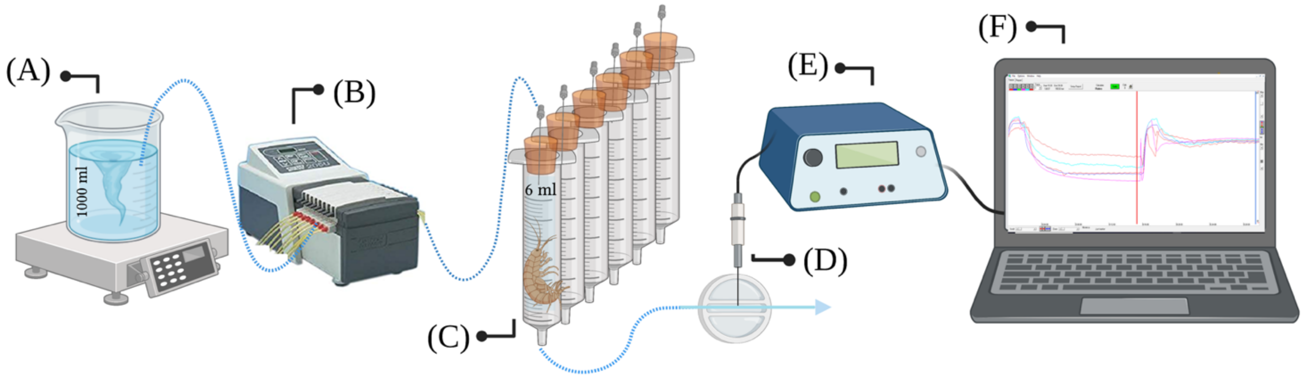

Fig. S1. Diagram of the Strathkelvin respirometry setup which consists of (A) a magnetic stirrer and a glass water tank, (B) a peristaltic pump, (C) respirometer chambers housing a single individual, (D) a Clark-type microelectrode, (E) a Strathkelvin oxymeter, (F) Strathkelvin software. Diagram created with BioRender.com, publication license AS2471MFU9.

Journal of Experimental Biology • Supplementary information

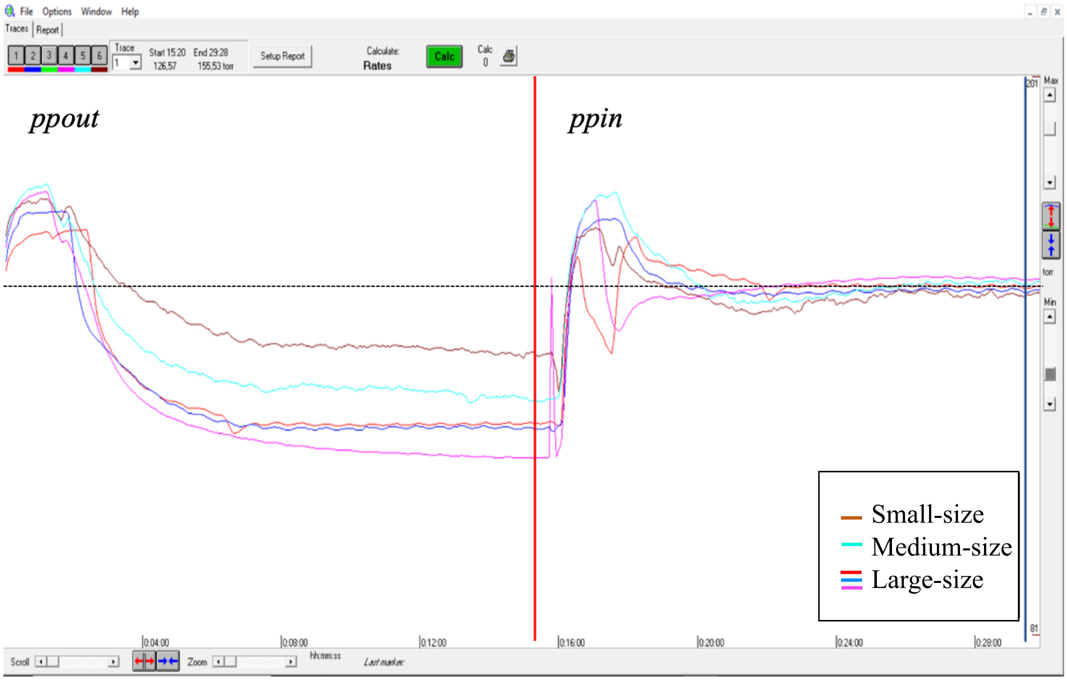

Fig. S2. Oxygen traces of five individuals of different masses. Brown = small, cyan = medium-sized and red, blue, fuchsia = large. Oxygen consumption curves in the presence of a specimen (*ppin*) and in the absence of specimens (*ppout*).

Journal of Experimental Biology • Supplementary information

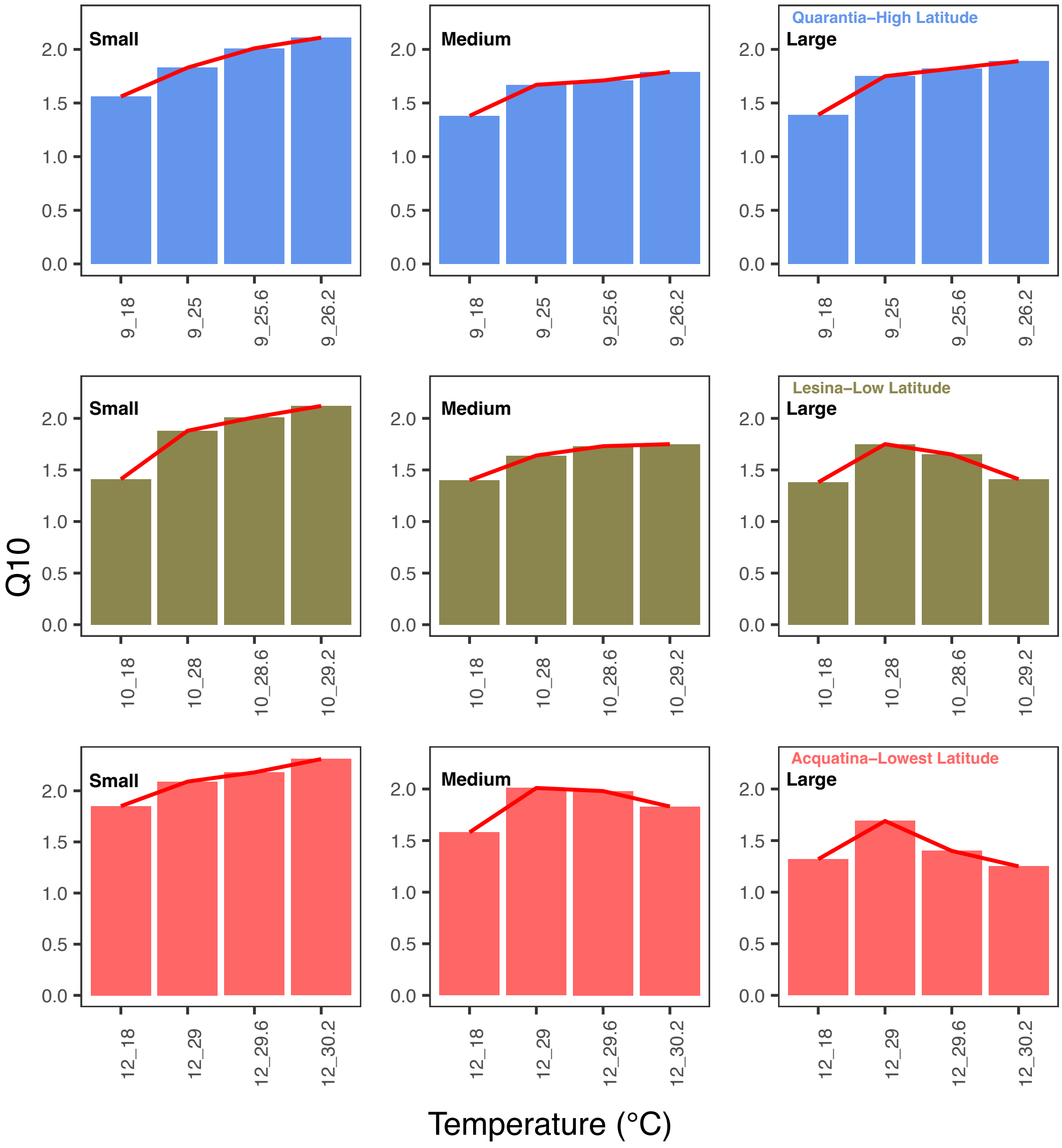

Fig. S3. Summary of temperature coefficients (Q10) for each temperature with respect to the minimum temperature, at each body-mass class and collection site.

Journal of Experimental Biology • Supplementary information
